# Agreement between a single-item measure of anxiety and depression and the Hospital Anxiety and Depression Scale: A cross-sectional study

**DOI:** 10.1371/journal.pone.0210111

**Published:** 2019-01-04

**Authors:** Heidi Turon, Mariko Carey, Allison Boyes, Bree Hobden, Sophie Dilworth, Rob Sanson-Fisher

**Affiliations:** 1 Health Behaviour Research Collaborative, University of Newcastle, Callaghan, New South Wales, Australia; 2 Priority Research Centre for Health Behaviour, University of Newcastle, Callaghan, New South Wales, Australia; 3 Hunter Medical Research Institute, New Lambton Heights, New South Wales, Australia; Leiden University, NETHERLANDS

## Abstract

Anxiety and depression can be heightened among individuals living with chronic diseases. Identifying these individuals is necessary for ensuring they are provided with adequate support. Traditional tools such as clinical interviews or symptom checklists are not always feasible to implement in practice. Robust single-item questions may be a useful alternative. This study aimed to measure agreement, sensitivity, specificity, positive predictive value and negative predictive value of a single-item question about anxiety and depression compared to the widely used Hospital Anxiety and Depression Scale (HADS). A cross-sectional survey of 2,811 people with cancer attending 19 treatment centres in Australia. Patients were approached in the waiting room prior to an outpatient clinic appointment and invited to complete a pen and paper survey. Participants completed the HADS as well as 2 single-items asking if they have felt anxious or depressed in the last week. The single-items for anxiety and depression each demonstrated moderate levels of sensitivity (0.78 for anxiety; 0.63 for depression) and specificity (0.75 for anxiety; 0.84 for depression) against the relevant HADS subscale. Positive predictive values were moderate (0.53 for anxiety and 0.52 for depression) while negative predictive values were high for both single-item questions (0.90 for anxiety and 0.89 for depression). The single-item measures of anxiety and depression may be useful to rule out individuals who do not require further psychological assessment or intervention for anxiety and depression. Further research is needed to explore whether these findings generalise to other chronic diseases.

## Introduction

### Identifying depression and anxiety among those with a chronic disease is difficult in clinical practice

The prevalence of depression and anxiety amongst people with a chronic disease (including stroke, cancer, heart disease and diabetes) is higher than in the general population[1]. While it is recommended across several disease groups that these conditions be identified, monitored and managed [2–4], this is often difficult to perform routinely within busy clinical settings. For instance, the Structured Clinical Interview for the Diagnostic and Statistical Manual of Mental Disorders is the gold standard for diagnosing anxiety and depression, and provides a comprehensive assessment of the severity of symptoms and their impact on functioning [5]. However, this interview is rarely used in clinical practice outside of the mental health sector, as it is time intensive to administer and requires specialised skills and training [6]. Furthermore, there is often an overlap in somatic symptoms between symptoms of chronic diseases and depression or anxiety, which may include fatigue, sleep problems, and poor appetite [7, 8]. This can make it difficult for health care providers to identify if symptoms of depression or anxiety are occurring, as opposed to symptoms of the chronic disease, or indeed side effects of medication. Previous research has found health care providers also have a tendency to under-detect symptoms of anxiety and/or depression when making unassisted judgements across a number of chronic diseases [9–12].

### Patient self-report assessments can assist in identifying depression and anxiety amongst those with chronic diseases

Self-report instruments can serve an important role in routinely assessing psychological symptoms across a variety of clinical applications. For example, these measures can be used to determine which patients might require further evaluation, or to measure the severity of distress so that appropriate levels of support can be provided [4, 13]. They may also be used to monitor outcomes of those receiving psychosocial interventions to determine whether the intervention is successful in reducing the severity of symptoms [14, 15]. There are a number of brief tools for assessing psychological wellbeing which have been recommended for routine use in clinical settings [16], including the Hospital Anxiety and Depression Scale (HADS) [17, 18]. These tools typically have moderate to high sensitivity and specificity compared to gold standard clinical interview, and usually range in length from 5 to 14 items [19, 20]. However, these tools may still be poorly implemented in clinical practice due to lack of time to administer the tools in a busy clinic setting, and lack of staff training in tool administration, scoring and interpretation [21, 22]. Given the barriers to using clinical interviews and brief screening tools, there has been increasing interest in utilising ultra-short tools to identify those who may have anxiety or depression in clinical settings. Ultra-short tools, such as the Distress Thermometer [23] or the Patient Health Questionnaire-2 (PHQ-2)[24], typically consist of between 1 and 4 items and can be rapidly completed by patients [19]. Studies have suggested that ultra-short tools may have some clinical utility in accurately screening out non-cases [19, 25, 26]. Although much of the research to date has focused on the Distress Thermometer, the wording of this tool is not ideal for use in all clinical contexts [27]. The Distress Thermometer focuses on a singular broad construct, i.e “distress” and has been shown to have only modest performance when anxiety and depression are the targets for assessment [28, 29]. Therefore, there is value in exploring the performance of single-item questions which focus on the constructs of anxiety and depression within chronic disease groups.

### Examining single-item questions within an oncology setting

It is estimated that approximately 16% of people with cancer experience depression, and approximately 10% experience anxiety [30]. Emotional distress may be associated with poor treatment adherence and reduced quality of life [31, 32]. A key feature of quality cancer care is the provision of psychosocial care that aims to reduce the impact of cancer on emotional wellbeing [33, 34]. Although the utility of single-item measures of anxiety and depression are starting to be explored in other chronic diseases [35, 36], most of the studies examining single-item measures, and indeed anxiety and depression screening, have been conducted in oncology settings [37, 38]. Thus we believe cancer patients, in the first instance, are an appropriate chronic disease population to test newly developed single item measures to allow for performance comparisons. When considering the potential clinical utility of a new screening measure, several metrics are useful to consider. Agreement examines how frequently the outcome of the measure agrees with the outcome of alternative measure. Sensitivity and specificity refer to the accuracy of the test to correctly identify those who do and do not have a particular condition. Positive predictive value (PPV) and negative predictive value (NPV) are related to the prevalence of a condition and provide insight into the likelihood an individual has (or does not have) a specific condition given a positive (or negative) test result[39]. The aim of this study was therefore, to examine the level of agreement, sensitivity, specificity, PPV and NPV of a single-item question for: (i) anxiety compared with a HADS anxiety score ≥8; and (ii) depression compared with a HADS depression score ≥8. The impact of gender on accuracy of the single-item measures was also explored, given previous studies suggesting that gender may influence reported distress [40, 41].

## Method

### Design and setting

This work was part of a larger cross-sectional study examining individual, social, and disease-related factors on psychological wellbeing in people diagnosed with cancer. It was conducted in 19 hospitals across six Australians states. This included 13 medical oncology clinics and nine haematology clinics, some of which were co-located within the same hospital. Sixteen hospitals were located in major metropolitan areas, while 3 were located in major regional areas. Participants were recruited between October 2012 and October 2014.

### Participants

Eligible participants were: (i) those with a confirmed diagnosis of cancer (any type or stage); (ii) attending an outpatient oncology appointment at one of the participating clinics; (iii) aged 18 years or older; (iv) able to read and understand English; (v) judged by clinic staff to be physically and psychologically capable of completing the questionnaire; and (vi) attending their second or subsequent appointment at the clinic.

### Procedure

Ethics approval for the study was gained from the University of Newcastle Human Research Ethics Committee (H-2011-0312, H-2010-1324), as well as all applicable hospital/health service ethics committees. Clinic staff identified potentially eligible patients who were then approached by a research assistant prior to their appointment and invited to participate in the study. Informed written consent was obtained from all participants.

Participants were asked to complete a self-report pen-and-paper questionnaire containing items about their sociodemographic characteristics and emotional wellbeing. Participants could complete the questionnaire while waiting for their appointment or take it home and post it back to the research team in the reply paid envelope provided. Non-responders were sent up to two reminders at two-weekly intervals. The age and gender of non-consenters were recorded so that any evidence of consent bias could be examined.

### Measures

#### Demographic, disease and treatment characteristics

Demographic items assessed gender, age, home postcode, Aboriginal or Torres Strait Islander background, marital status, education level, country of birth, and employment status. Disease and treatment items assessed cancer type, cancer stage at diagnosis, time since diagnosis, and treatments received and planned.

#### Hospital Anxiety and Depression Scale (HADS)

The HADS [17] is a 14 item self-report questionnaire comprising of two subscales measuring symptoms of depression (HADS-D, 7-items) and symptoms of anxiety (HADS-A, 7-items) over the last week. Items are worded to direct participants to reflect on how frequently they have experienced common symptoms including restlessness, enjoyment of everyday things, and worry. Scoring for each item ranges from 0 to 3, with a maximum possible score of 21 for each subscale. A cut-off score of 8 or more for each subscale is recommended for identifying ‘possible caseness’ of anxiety and depression [17]. The HADS has demonstrated reliability and validity among people with cancer [42]. The HADS was selected as a proxy for the “gold-standard” in this study as it is commonly used and accepted amongst both clinicians and researchers as a screening tool for depression and anxiety in patients with chronic diseases [43, 44] including cancer [45–47]. It was also designed to exclude references to somatic symptoms, which make it suitable for use with chronic disease populations [48].

#### Single-items for depression and anxiety

Participants were asked: Over the past week have you: a) Felt anxious? (Response options: Yes/No); and b) Felt depressed? (Response options: Yes/No). The correlation between the two items was 0.477.

### Data analysis

Frequencies and percentages were calculated for all demographic and disease characteristics. HADS subscale scores were calculated for those participants with no more than 1 item missing from the subscale. The mean score for the remaining subscale items was imputed for the missing item, if needed. Agreement was calculated as the sum of: a) the proportion of participants who were classified as anxious/depressed by HADS and also self-reported being anxious/depressed; and b) the proportion of participants who were classified as not anxious/depressed by HADS and also self-reported being not anxious/depressed. Sensitivity, specificity, PPV and NPV were calculated using the HADS as the gold standard (actual) and self-report items as the test classifier. Sensitivity refers to the proportion of people answering “yes” to the single-item question who were classified as cases on the corresponding HADS subscale. Specificity refers to the proportion of people responding “no” to the single-item question who were classified as non-cases on the corresponding HADS subscale. PPV refers to the probability of someone being anxious (or depressed), given they answered “yes” to the single-item question. NPV refers to the probability of someone not being anxious (or depressed) given that they answered “no” to the single-item question. A HADS-D and HADS-A cutoff score of 8 or higher was used to define “caseness”, per the original authors recommendation [17]. To explore whether the self-report measure differed by gender, a logistic regression of HADS measures on self-report measure adjusting for age and gender was performed. An interaction term for self-report by gender was initially included to determine if there was a statistically significant difference by gender. If the interaction term was not significant it was removed from the model. From the resulting model, comparison of accuracy of the self-report measure by gender was done by comparing AUROCs (plots of sensitivity vs 1-specificity) for each gender [49]. Statistical analyses were programmed using Stata v14.0 (StataCorp Ltd, College Station, TX).

## Results

### Sample

A total of 4,233 eligible patients were approached about the study of which 3,472 (82%) consented to participate. Of consenting patients, 2,811 (81%) completed the questionnaire and were included in the analyses. There were no statistically significant differences in the age or gender of consenters and non-consenters (p>.05). A slight majority of the included sample were female (56%) and aged over 60 (56%). Breast was the most common cancer type (26%), however, a large portion selected ‘other’ cancer (29%). The characteristics of the study sample are shown in Table 1.

**Table 1:** Demographic and disease characteristics of the study sample (N = 2,811).

Characteristic		Total n (%)
Gender	Male	1,216 (43%)
	Female	1,588 (56%)
Age at questionnaire completion	18–49	516 (18%)
	50–59	684 (24%)
	60–69	837 (30%)
	70+	730 (26%)
Type of cancer	Breast	727 (26%)
	Colorectal	332 (12%)
	Non-Hodgkin’s lymphoma	249 (8.86%)
	Leukaemia (all types)	234 (8.32%)
	Myeloma	174 (6.19%)
	Lung	171 (6.08%)
	Other	807 (29%)
Time since diagnosis	0-12months	1,195 (43%)
	1–2 years	475 (17%)
	2+ years	1,106 (39%)
Received surgery	Yes	1,622 (58%)
	No	1,146 (41%)
Received radiotherapy	Yes	1,166 (41%)
	No	1,500 (53%)
Chemotherapy	Yes, have received or are planning on receiving	2,330 (83%)
	No, have not received and are not planning on receiving	422 (15%)
Marital status	Married or partner	1,824 (65%)
	Single, divorced, separated or widowed	950 (34%)
Education completed	High school or below	1351 (48%)
	Vocational, University, other	1410 (50%)
Employment status	Paid employment	888 (32%)
	Not in labour force, unemployed, other	1880 (67%)
Place of birth	Australia	1936 (69%)
	Other than Australia	802 (29%)

Note: Totals may not add up to 100% due to missing values.

### Agreement between HADS and single-item for identifying possible cases of anxiety and depression

Anxiety: Agreement between single-item and HADS classified anxiety is displayed in Table 2. Overall, there was a 76% observed agreement between the single-item and HADS-A for possible anxiety. The single-item anxiety measure had a sensitivity of 0.78 (95%CI = 0.75–0.81) and a specificity of 0.75 (95% CI = 0.73–0.77). The positive predictive value (PPV) of the single-item anxiety measure was 0.53 (95% CI = 0.50–0.56), and the negative predictive value (NPV) was 0.90 (95% CI = 0.89–0.92). The interaction of self-reported anxiety and gender was not statistically significant (p = 0.245) and was removed from the model. The resulting model AUROC was 0.79 (95%CI: 0.77–0.81). AUROCs by gender were 0.79 (95% CI 0.76–0.82) for males and 0.78 (95%CI 0.75–0.80) for females and these were not statistically significantly different (p = 0.4860).

**Table 2:** Agreement between single-item and HADS classified anxiety.

	Possible anxiety (HADS-A)		Total
Anxiety (single-item)	No	Yes	
No	1,477 (55%)	158 (6%)	1,635 (60%)
Yes	499 (18%)	570 (21%)	1,069 (40%)
Total	1,976 (73%)	728 (27%)	2,704 (100%)

Depression: Agreement between single-item and HADS classified depression is displayed in Table 3. Overall, there was an 80% observed agreement between the single-item and HADS-D for possible depression. The single-item depression measure had a sensitivity of 0.63 (95%CI = 0.59–0.67) and a specificity of 0.84 (95% CI = 0.83–0.86). The positive predictive value (PPV) of the single-item anxiety measure was 0.52 (95% CI = 0.48–0.56), and the negative predictive value (NPV) was 0.89 (95% CI = 0.88–0.91). The interaction of self-reported depression and gender was not statistically significant (p = 0.442) and was removed from the model. The resulting model AUROC was 0.77 (95%CI: 0.74–0.79). AUROCs by gender were 0.75 (95% CI 0.71–0.79) for males and 0.77 (95%CI 0.73–0.80) for females and these were not statistically significantly different (p = 0.5500).

**Table 3:** Agreement between single-item and HADS classified depression.

	Possible depression (HADS-D)		Total
Depression (single-item)	No	Yes	
No	1,771 (66%)	214 (8%)	1,985 (74%)
Yes	329 (12%)	358 (13%)	687 (26%)
Total	2,100 (79%)	572 (21%)	2,672 (100%)

## Discussion

Accuracy of a single-item measure of anxiety and a single-item measure of depression, compared to the HADS subscales for anxiety and depression, were examined in this study amongst a large sample of people diagnosed with cancer. The pattern of results was similar for both the anxiety and depression subscales. The single-item anxiety measure demonstrated moderate levels of sensitivity and specificity with the HADS-A. Of every 100 people identified as having possible anxiety by the HADS-A, the single-item identified 78 of these. Conversely, of every 100 people identified as not anxious as measured by the HADS-A, 75 were identified as not anxious by the single-item. A previous meta-analysis examined four studies comparing the sensitivity and specificity of the Distress Thermometer to HADS-A [26] and found a pooled sensitivity of 0.77 and pooled specificity of 0.56. The single-item anxiety measure used in our study, therefore, performed slightly better than the Distress Thermometer among a sample of cancer patients, particularly for specificity.

The single-item depression measure had slightly lower sensitivity compared to the anxiety item, identifying 63 out of every 100 people that the HADS-D identified as possibly depressed. The specificity of the depression item was slightly higher than the anxiety item. Of every 100 people identified as not depressed as measured by the HADS–D, the single-item depression measure identified 84 of these as similarly not depressed. Compared to the findings of a meta-analysis of ultra-short screening methods for depression (including the distress thermometer, as well as some single or dual question items), we found a lower level of sensitivity (0.63 versus 0.78 in the meta-analysis), but a higher level of specificity (0.84 versus 0.67 in the meta-analysis) [26]. When examining single studies included in the meta-analysis there appears to be a trend for studies using the distress thermometer to have higher sensitivity and lower specificity, than studies using other ultrashort measures (such as 1 or 2 items about depression). However, this finding is complicated by the choice of gold-standard comparator, which was more commonly HADS or another scale for the distress thermometer, whereas the other ultrashort measures were mostly compared with a structured clinical interview.

Other potential reasons contributing to this difference in findings may include differences in the setting under investigation, i.e. palliative care; or a limited sample size in previous studies.

We found that the PPVs and NPVs were similar for both single-items compared to the HADS subscales. The single-items had moderate PPVs, indicating that just over half of those who answered “Yes” to the single-item had possible anxiety or depression. Comparatively, the value of the NPVs were high. Most who answered “No” to the single-items (89% for depression and 90% for anxiety), were not anxious or depressed according to HADS. These findings echo results from Mitchell et al. [19, 26, 50] demonstrating that across a variety of ultra-short tools, ability to identify potential cases of anxiety or depression is modest, and cannot be solely relied upon to detect probable cases. These tools have potential validity, however, in identifying non-cases of anxiety and/or depression.

We did not find any differences in accuracy for the single-item measures based on gender. This suggests that the single-item depression and anxiety questions were not better proxy measures of anxiety or depression for females as opposed to males (or vice versa) and supports the use of the single-item measures for both females and males in a clinical environment.

### Clinical implications

The findings suggest that use of a single-item as a screening tool for anxiety and depression amongst people who have cancer may have value in identifying non-cases. There could be benefit in a 2 stage assessment approach, whereby single-items could be used as a first step in identifying people with possible anxiety and depression. Such an approach represents a relatively low burden method for quickly identifying people who are not experiencing these symptoms and are unlikely to require additional psychosocial support. Those who answer “yes” to feeling anxious or depressed could be referred for a more detailed assessment and then, based on this assessment, provided with the necessary support. Mitchell and Coyne [51] argue that when applying a second step in the screening process, particularly for a condition with relatively low prevalence (such as depression), and a tool with high specificity is used in the first step, a tool with high sensitivity should be selected for the second step in order to have the greatest gains in overall accuracy. Screening tools with higher sensitivity, include for example, the Beck Depression Inventory-II [25, 52]. Alternatively, dependent on clinic resources, this may be a formal diagnostic interview. Individuals who answer “yes” but do not meet diagnostic criteria could also be provided with self-management resources and re-assessed at a later point.

The HADS takes longer to administer than the single item measures to complete, i.e. approximately 2–5 minutes to complete the HADS [53] compared to less than 30 seconds for the single item measures. The HADS also requires additional time and expertise to be scored and interpreted, hence the advantage of ultra-short tools. It is possible that repeated positive responses on a single item (for example, at two consecutive consultations) may have greater utility in identifying clinically relevant distress than the single item assessment on a single occasion. This may be a focus of future research, particularly for settings and contexts where the implementation of longer assessments is unlikely to be feasible.

### Study limitations

Although this study included a large and diverse sample of people with cancer attending outpatient clinics from multiple treatment centres, the survey was not administered to inpatients, or people who were acutely unwell on the day of recruitment. Therefore, results may not be generalisable to these groups. As with all self-report measures, the likelihood of response bias should be considered, however, we attempted to minimise this by ensuring the survey was de-identified and individual responses were not shared with treatment providers. While a large proportion of participants completed the survey prior to the consultation, due to variations in waiting times across clinics, some participants completed the survey post-consultation. This is a common but unfortunate pragmatic issue when recruiting participants in outpatient clinics. The consultation itself thus presents a possible source of bias in the results. An additional limitation of the single-item for self-identifying feeling anxious or depressed in the past week is the individual variance in interpreting this question. The simplicity of the yes/no response scale does not capture the severity of an individual’s symptoms. It should also be acknowledged that although the HADS has been extensively validated, debate remains about appropriate cut points [54]. As such, we elected to use the threshold recommended by the authors of the tool for identifying possible cases of anxiety and/or depression.

### Conclusion

This large, multisite cross-sectional study of people with cancer attending outpatient clinics revealed that a single-item regarding anxiety or depression had relatively high levels of specificity in detecting non-cases when compared to the HADS, but poor levels of sensitivity. This finding is concordant with previous literature examining the accuracy of ultra-short tools. The high NPVs in this study supports the notion that a single-item measure has potential utility in ruling out those who are likely to require psychosocial intervention. Further research is needed to determine whether these results can be replicated for other chronic diseases, however the single-item approach has the potential to provide a quick and pragmatic option for healthcare providers wishing to routinely screen for anxiety and depression in cancer patients.
